# Prediction of extranodal extension in head and neck squamous cell carcinoma by CT images using an evolutionary learning model

**DOI:** 10.1186/s40644-023-00601-7

**Published:** 2023-09-12

**Authors:** Tzu-Ting Huang, Yi-Chen Lin, Chia-Heng Yen, Jui Lan, Chiun-Chieh Yu, Wei-Che Lin, Yueh-Shng Chen, Cheng-Kang Wang, Eng-Yen Huang, Shinn-Ying Ho

**Affiliations:** 1 Department of Radiation Oncology and Proton & Radiation Therapy Center, Kaohsiung Chang Gung Memorial Hospital, Chang Gung University College of Medicine, No. 129, Dapi Road, Niaosong District, Kaohsiung, Taiwan; 2 Institute of Computer Science and Engineering, National Yang Ming Chiao Tung University, No. 1001 University Road, Hsinchu, Taiwan; 3 Institute of Bioinformatics and Systems Biology, National Yang Ming Chiao Tung University, No. 75 Po- Ai Street, Hsinchu, Taiwan; 4 Department of Anatomic Pathology, Kaohsiung Chang Gung Memorial Hospital, Chang Gung University College of Medicine, No. 123, Dapi Road, Niaosong District, Kaohsiung, Taiwan; 5 Department of Diagnostic Radiology, Kaohsiung Chang Gung Memorial Hospital, Chang Gung University College of Medicine, No. 123, Dapi Road, Niaosong District, Kaohsiung, Taiwan; 6 School of Medicine, College of Medicine, National Sun Yat-sen University, No. 70, Lienhai Rd, 80424 Kaohsiung, Taiwan; 7 Department of Biological Science and Technology, National Yang Ming Chiao Tung University, No. 1001 University Road, Hsinchu, Taiwan; 8 Center for Intelligent Drug Systems and Smart Bio-Devices (IDS 2 B), National Yang Ming Chiao Tung University, No. 75 Po-Ai Street, Hsinchu, Taiwan; 9 College of Health Sciences, Kaohsiung Medical University, No. 100, Shih-Chuan 1st Road, Sanmin District, Kaohsiung, Taiwan

**Keywords:** Head and neck squamous cell carcinoma, Extranodal extension, Radiomics, Evolutionary learning, Artificial intelligence

## Abstract

**Background:**

Extranodal extension (ENE) in head and neck squamous cell carcinoma (HNSCC) correlates to poor prognoses and influences treatment strategies. Deep learning may yield promising performance of predicting ENE in HNSCC but lack of transparency and interpretability. This work proposes an evolutionary learning method, called EL-ENE, to establish a more interpretable ENE prediction model for aiding clinical diagnosis.

**Methods:**

There were 364 HNSCC patients who underwent neck lymph node (LN) dissection with pre-operative contrast-enhanced computerized tomography images. All the 778 LNs were divided into training and test sets with the ratio 8:2. EL-ENE uses an inheritable bi-objective combinatorial genetic algorithm for optimal feature selection and parameter setting of support vector machine. The diagnostic performances of the ENE prediction model and radiologists were compared using independent test datasets.

**Results:**

The EL-ENE model achieved the test accuracy of 80.00%, sensitivity of 81.13%, and specificity of 79.44% for ENE detection. The three radiologists achieved the mean diagnostic accuracy of 70.4%, sensitivity of 75.6%, and specificity of 67.9%. The features of gray-level texture and 3D morphology of LNs played essential roles in predicting ENE.

**Conclusions:**

The EL-ENE method provided an accurate, comprehensible, and robust model to predict ENE in HNSCC with interpretable radiomic features for expanding clinical knowledge. The proposed transparent prediction models are more trustworthy and may increase their acceptance in daily clinical practice.

**Supplementary Information:**

The online version contains supplementary material available at 10.1186/s40644-023-00601-7.

## Introduction

Extranodal extension (ENE) is a pathological diagnosis defined by the College of American Pathologists lip and oral cavity cancer protocol as “extension of metastatic tumor, present within the confines of the lymph node (LN), through the LN capsule into the surrounding connective tissue, with or without associated stromal reaction” [1]. ENE is a poor prognostic factor associated with increased locoregional failure, distant metastases, and reduced overall survival in patients with head and neck squamous cell carcinoma (HNSCC) [2–4].

The presence of ENE is critical in clinical decision-making. For patients with ENE-positive HNSCC, concurrent chemoradiotherapy may yield similar treatment outcomes to patients receiving surgery followed by adjuvant chemoradiation, while providing fewer treatment-related acute and late toxicities, and lower healthcare costs [5–8]. Therefore, developing an accurate, robust, and trustworthy prediction model to distinguish the ENE status before the definitive treatment is important to guide the best therapy for HNSCC patients.

Contrast-enhanced computed tomography (CT) scan is the most widely used method to predict ENE status for HNSCC patients in clinical practice. However, the literature revealed that this method has limited diagnostic performance, with reported sensitivity ranging from 43.7 to 69% and the area under the receiver operating characteristic curve (AUC) ranging from 0.6 to 0.69 [9–13]. Furthermore, high inter-observer variability is also reported [9, 11–13].

To improve the diagnostic performance of ENE by CT scanning, two studies applied deep learning methods to establish prediction models for ENE detection [14, 15]. Both studies showed excellent results with AUC of 0.91 and 0.82 for ENE prediction. Although deep learning models yield attractive results, these models often work as black-boxes with limited transparency and interpretability [16]. It is difficult for clinicians to correlate the results of these deep learning models with known radiomic features of ENE.

Identification of effective radiomic features plays a vital role in advancing prediction performance and providing interpretability associated with clinical knowledge. Lee et al. proposed an evolutionary learning (EL) method for establishing clinical-radiomic models to predict the early recurrence of hepatocellular carcinoma after resection, better than other well-known machine learning (ML) derived models [17]. This EL method aims to optimize the feature selection and model parameters in establishing ML models.

In this work, we use the novel EL approach to identifying a set of interpretable radiomic features. The proposed method EL-ENE uses the inheritable bi-objective combinatorial genetic algorithm (IBCGA) [18] with an intelligent evolutionary algorithm (IEA) [19] for optimal feature selection and parameter setting of support vector machine (SVM) to establish an interpretable model for predicting ENE by CT scanning.

## Materials and methods

### Patient selection, image acquisition, and characteristics

The medical records of consecutive patients with histologically proven HNSCC from 1 to 2009 to 31 October 2017 were reviewed retrospectively. Three hundred and sixty-four HNSCC patients who underwent neck LN dissection with preoperative contrast-enhanced diagnostic head and neck CT scans were enrolled. Exclusion criteria included previous neck surgery, preoperative chemotherapy/chemoradiotherapy, LN short axis < 1 cm on CT images, and the time between staging CT to LN dissection over 6 weeks. The Institutional Review Board of our institution approved this study (201801181B0/201801181B0C501/201801181B0C601).

The head and neck CT scans were performed on a 64-channel scanner (Aquilion 64, Toshiba Medical Systems, Tokyo, Japan), 80-channel scanner (Aquilion Prime, Canon Medical Systems, Otawara, Japan) or 256-channel scanner (Siemens Healthcare AG, Erlangen, Germany) with the following parameters: tube current 100–550 mAs; voltage 120 kVp; gantry rotation time 0.5 s; pitch 0.969 mm/rotation; detector collimation 80 × 0.5 mm; field of view 22 cm; and 3 mm axial reconstruction thickness. The CT images extend from the upper orbital rim through the upper thorax. Enhanced images were obtained 60 s after intravenous injection of 1.0 mL/kg CT contrast (Omnipaque 350, GE Healthcare, Princeton, New Jersey) at a rate of 2.0 mL/second. The CT scans were reviewed on a commercial Picture Archiving and Communication System (PACS) workstation. (Centricity RA 1000; GE Healthcare, Chicago, IL, USA).

All pathology specimens were collected and reviewed by one head and neck pathologist (J. Lan) to avoid interobserver variation. ENE was defined as tumor infiltrating from the capsule of a metastatic LN [1]. For each LN, a one-to-one matching between the pre-operative CT images and the pathology report was obtained according to the LN’s laterality, anatomical level, and nodal size. If there were more than one LN with a similar size at the same region on the CT image where a definite correlation could not be derived, these LNs were not included in the study. The regions of interest (ROIs) were delineated manually at the edge of LNs on each slice in the axial plane and were recorded in the RT structure set (RTSS) label file. The segmentation process was done by one radiation oncologist (T.T. Huang) to ensure contouring consistency.

The CT images used included 364 patients with 778 3D LN images. The dataset contained 375 normal LNs, 139 metastasis LNs, and 264 ENE LNs. The CT image format was Digital Imaging and Communications in Medicine (DICOM), and the size was 512*512 pixels. Among them, 22 patients had synchronous head and neck cancers with 391 primary sites. The most common primary disease site was the oral cavity. Only 2.2% of patients in the cohort had positive p16 status. The detailed patient characteristics are listed in Table 1.

**Table 1:** Demographics (364 patients; 391 primary sites; 778 LNs)

Characteristic	Value
Age	
Mean	54.4 ± 10.8
Gender	
Male	333 (91.5%)
Female	31 (8.5%)
Primary cancer site	
Oral cavity	289 (73.9%)
Oropharynx	47 (12.0%)
Hypopharynx	36 (9.2%)
Larynx	17 (4.4%)
Salivary glands	2 (0.5%)
Pathological T stage	
T1	80 (20.5%)
T2	106 (27.1%)
T3	35 (9.0%)
T4	162 (41.4%)
Biopsy only	8 (2.0%)
Pathological N stage	
N0	134 (36.8%)
N1	62 (17.0%)
N2	164 (45.1%)
N3	4 (1.1%)
p16 status	
Positive	8 (2.2%)
Negative	337 (92.6%)
Unknown	19 (5.2%)
LN status	
Negative	375 (48.2%)
Metastatic with ENE(-)	139 (17.9%)
Metastatic with ENE(+)	264 (33.9%)

The 778 3D LN images were divided into a training set and a test set by the approximate ratio 8:2. The training set had 618 LNs of 314 patients, including 296 negative LNs, 111 metastasis LNs, and 211 ENE LNs. The test set had 160 LNs of 50 patients, including 79 negative LNs, 28 metastasis LNs, and 53 ENE LNs.

### The proposed method EL-ENE

**Fig. 1:**
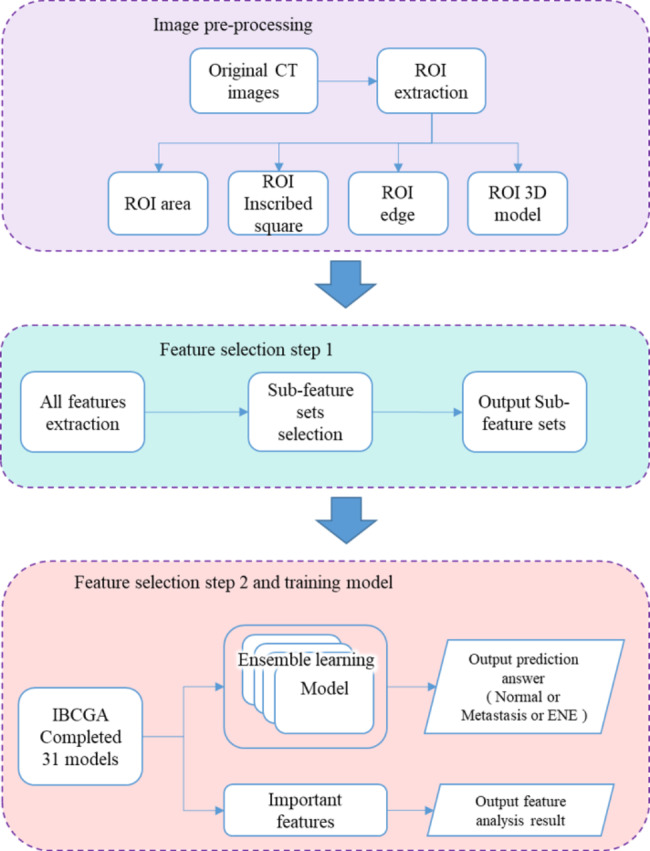
The flowchart of EL-ENE including image pre-processing, feature extraction, feature selection, and ensemble classifier of support vector machine

The proposed method EL-ENE used an evolutionary learning approach to identifying a small set of radiomics features while maximizing the prediction accuracy. Figure 1 shows the flowchart of EL-ENE, including image pre-processing, feature extraction, feature selection, and ensemble classifier of SVM [20].

### Image pre-processing

The image pre-processing for extracting ROIs includes three main tasks, including (1) extraction of the volume of Interest (VOIs), (2) superimposition of CT image and RTSS annotation, and (3) extraction of ROIs from the DICOM images. First, the new Window Center was adjusted using Window Center, Rescale Slope, and Rescale Intercept in the DICOM file header. Then, the VOI was calculated using the new Window Center and Window Width, and was normalized into the range of [0, 255]. The coordinate information of the ROI was recorded in the RTSS annotation file. The normalized CT image was superimposed with the ROI coordinate, and the desired ROI contour position map in the DICOM file was obtained.

The boundary information of LNs is highly associated with the ENE. To ensure that the contours detected were complete, we extracted the accurate ROI using morphological operations, including dilation, fill, and erosion. Finally, the designed mask using the morphological operations was operated on the calibrated CT images, and the tomographic images of the LN sections were extracted. The Imdilate, imfill, and imerode functions in the Matlab tool were used to extract the ROI boundary. In addition, we also extracted the ROI inscribed square and the ROI contour information for subsequent image analysis, e.g., feature extraction from the gray level change inside and outside the ROIboundary.

### Feature extraction

We extracted gray level, geometric, morphological, and texture features from CT images of LNs as candidate features. There were 460 candidate features, which were categorized into six types of features with 26 feature subsets (Table 2). The six types were Gray-Level Co-occurrence Matrix (GLCM), Gray-Level Size Zone Matrix (GLSZM), Gray-Level, LN morphology, LN boundary, and Invariant moment.

**Table 2:** The 26 subsets belonging to six feature types

	Feature type	Subset of features	Number of features
1	Gray-Level Co-occurrence Matrix	Cluster Shade	20
		Cluster Proximity	20
		Contrast	20
		Correlation	20
		Different Entropy	20
		Different Variance	20
		Dissimilarity	20
		Energy	20
		Entropy	20
		Homogeneity Normalized	20
		Homogeneity	20
		InfoCorrelation1	20
		InfoCorrelation2	20
		Max Probability	20
		Sum Average	20
		Sum Entropy	20
		Sum of variance	20
		Variance	20
2	Gray-Level Size Zone Matrix	GLSZM	11
3	Gray-Level	Gray level	11
4	LN morphology	3D Morphology	29
		2D Morphology	24
5	LN boundary	Edge 3	6
		Edge 5	6
		Edge 10	6
6	Invariant moment	Invariant moment	7

The GLCM, GLSZM, and Invariant moment features were extracted from the largest inscribed square of the largest ROI section in the LN. The GLCM features reflect the texture distribution by counting the gray level changes between the two pixels at the space in various angles and distances. GLCM features contain four types of gray quantitative features [21] and 14 types of Haralick features [22], including Cluster Shade, Cluster Proximity, Contrast, Correlation, Different Entropy, Different Variance, Dissimilarity, Energy, Entropy, Homogeneity Normalized, Homogeneity, InfoCorrelation1, InfoCorrelation2, Max Probability, Sum Average, Sum Entropy, Sum of variance, and Variance. Each feature type contains 20 features calculated from 20 GLCMs with different angles and distances. The parameter range of GLCM was set to 16 grey levels, the directions were 0 °, 45 °, 90, and 135 °, and the distances were integers from 1 to 5.

GLSZM calculated the change of gray levels in ROI by quantizing the gray area in the image [23]. Unlike GLCM, GLSZM calculated a matrix for the domains connected in all directions for the same gray levels, regardless of rotation and distance. The parameter of GLSZM for the gray level range was set to 16 levels, and 11 features can be obtained, including small area emphasis, large area emphasis, low intensity emphasis, high intensity emphasis, low intensity small area emphasis, low intensity large area emphasis, high intensity small area emphasis, high intensity large area emphasis, intensity variance, size zone variance, and zone%. Invariant moments are often used as optical character recognition and shape recognition features in images. Their moment invariance was not changed by the rotation, translation, and scaling of images [24, 25]. Through the second-order and third-order central moments, seven invariant moments were obtained as features.

Gray-Level and 2D LN morphology features were extracted from the largest ROI section in the LN. For the gray level features of ROI, they show the statistical analysis of the numerical changes of the gray levels in the ROI, including ten features such as Mean, Median, Variance, Standard deviation, Maximal of gray levels, Minimal of gray levels, Skewness, Kurtosis, Energy, and Entropy. The 2D morphological features are the surface configuration of image objects, which are essential in distinguishing LNs. 24 features were collected, including Area, Perimeter, Major Axis Length, Minor Axis Length, Orientation, Convexity, Convex Area, Convex Perimeter, Maximum radius, Bounding Box Area, Defects Ratio, Perimeter Area Ratio, Aspect Ratio, Bending Energy, Eccentricity, Equivalent Diameter, Solidity, Extent, Compactness, Rectangularity, Elongation, Roundness, Ellipiticty, and Sphericity.

3D LN morphology features were extracted from the 3D LN model. First, a series of LN CT images were stacked. Then, the height of the stacked 3D LN model was corrected by using the actual width of the pixel (e.g., 0.4680 mm) and slice thickness (e.g., 3 mm) recorded in DICOM head file. Finally, Delaunay triangulation was used to smooth the surface of interpolated LNs (using the interp3 function of Matlab). Twenty-nine features were collected, including Volume, Surface, Equivalent diameter, Extent, three Principal Axis Length, three Orientation, Eccentricity, Solidity, Convex volume, Convex surface, Convexity, Compactness, Rectangularity, Elongation, Roundness, Area volume ratio, three Aspect radius, Maximum radius, Bounding box volume, Ellipiticty, Defect ratio, Gaussian Curvature sum, and Mean Curvature sum.

The LN boundary was extracted from the ROI section in the LN. For boundary features, the gray level changes inside and outside the ROI area are related to whether the LNs expand outside the LNs. The Imdilate function of Matlab was used to extract the ROI boundary area, which is dilated with disc-shaped structural elements, considering the radius of the disc shape with 3, 5, and 10 pixels. For each ROI boundary area on CT images, 6 LN boundary features were extracted, including Mean inside the ROI, Mean outside the ROI, Standard variance inside the ROI, Standard variance outside the ROI, differences of Mean, and Standard variance between inside and outside the ROI.

### Feature selection

Due to the large number of candidate features, EL-ENE used a coarse-to-fine feature selection. The coarse step is to independently evaluate each of the 26 feature subsets using the classification accuracy of SVM in terms of 10-fold cross-validation (10-CV). For each feature subset, three SVM models were established to evaluate feature subsets. The three models predicted a LN as (1) normal or metastatic, (2) ENE or non-ENE, and (3) normal, metastatic, or ENE. For each model, we selected the top five feature subsets ranked by prediction accuracy. Experimental results revealed seven feature subsets with 89 features, including Sum of variance, GLSZM, Gray-Level, 3D Morphology, Edge 3, Edge 5, and Edge 10.

The fine step used an IBCGA [18, 19] cooperated with SVM to select a minimal number of features while maximizing prediction accuracy. IBCGA selects *m* form *n* (= 89 in this study) features and determines the parameter setting of SVM for training the prediction models. Since IBCGA is a non-deterministic algorithm, the obtained SVM models with identified features were not always the same. EL-ENE establishes an ensemble SVM classifier consisting of 31 SVM models with different sets of features that predicts LNs as normal, metastasis, or ENE.

### The customized IBCGA

EL-ENE uses an evolutionary learning approach to optimizing the system parameters in designing an interpretable classifier. The customized IBCGA algorithm was used to select a small number *m* from a large number *n* of radiomics features and determine two parameter values of the SVM model, cost *C* and γ of the kernel function.

The simultaneous optimization of feature selection and SVM parameters play a vital role in modeling. The *m* features can be ranked according to the prediction contribution using the main effect difference. Some applications of IEA and IBCGA in designing prediction models for biomedicine research can refer the studies [26–29].

In EL-ENE, the fitness function of IBCGA is to maximize the prediction accuracy of 10-CV on the training dataset. The best value of *m* was automatically determined belonging to the range [*r*_end_, *r*_start_]. The parameter settings of IBCGA were as follows: *N*_pop_=50, *P*_s_ = 1.0, *P*_c_ = 0.8, *P*_m_ = 0.05, *G*_max_ = 100, *r*_start_ =70, and *r*_end_=5. The main steps of IBCGA are as follows.

Step 1. Initialization: Generate a population of *N*_pop_ individuals randomly where each contains *r* = *r*_start_ selected features, (*n*-*r*_start_) unselected features, *C* and γ. *G* = 0.
Step 2. Evaluation: Evaluate all individuals using the fitness function.
Step 3. Selection: Select *P*_s_×*N*_pop_ individuals by a tournament selection method to form a mating pool.
Step 4. Crossover: Perform the orthogonal array crossover of IEA [19] on randomly selected *P*_c_×*N*_pop_ individuals.
Step 5. Mutation: Randomly select *P*_m_×*N*_pop_ individuals excluding the best one to mutate using a bit-swap operation.
Step 6. Termination test: Increase the number *G* by one. If *G* = *G*_max_, output the best individual in the population as *X*_r_, *G* = 0, and go to Step 7. Otherwise, go to Step 2.
Step 7. Inheritance: If *r* > *r*_end_, randomly mutate a binary gene from 1 to 0 for each individual, decrease the value of *r* by 1, and go to Step 2.
Step 8. Output: Let *X*_m_ with *m* selected features be the best individual among *X*_r_ where *r* = *r*_end_, *r*_end_+1, …, *r*_start_.

### Radiologists’ review protocol

Three neuroradiologists with more than 4 years of experience in head and neck imaging were recruited for assessing the status of ENE. LNs in the test data sets were annotated with serial numbers for review. Five radiomic features were applied for judging ENE presence, including irregular nodal enhancement, poorly defined nodal margins, infiltration of the adjacent fat plane, central necrosis, and matted nodes.

According to the 5 imaging features, the observers concluded the probability of ENE based on a 5-point rating score: 1, definitely not ENE; 2, likely not ENE; 3, equivocal ENE; 4, likely ENE and 5, definitely ENE. Scores 1 and 2 were deemed negative ENE while scores 3–5 were considered positive ENE [9, 11].

### Model evaluation and statistical analysis

The diagnostic performance of the prediction model was evaluated on the independent test data set using AUC, sensitivity, specificity, accuracy, positive and negative predictive values. The statistics were performed by R version 4.02 (The R Foundation for Statistical Computing, Vienna, Austria) and SPSS version 22.0 software (SPSS, Chicago, IL).

## Results

### Subset feature evaluation

Three types of prediction ability were tested for selecting the promising subset features as candidate ones for the feature selection of IBGGA. Table A1 listed the top five subset features with high 10-CV accuracy, which can distinguish the metastatic LNs. The top five subset features were Gray-level, Edge 10, Sum of variance, 3D morphology, and Edge 5. Table A2 listed the top five subset features which can distinguish the ENE LNs. The top five subset features were 3D morphology, Gray-level, Edge 5, GLSZM, and Edge 10. Table A3 listed the top five subset features which can distinguish three classes of LNs. The top five subset features were 3D morphology, Gray-level, Edge 3, Sum of variance, and Edge 10.

The 3D morphology, Gray-level, and Edge 10 were selected in three types of evaluations. The Sum of variance and Edge 5 were selected in two of them, and Edge 3 and GLSZM were selected once. The results show that morphology, gray level, and edge features were important in distinguishing the LN types. The seven subsets with 89 features were selected as the input feature for the EL-ENE method.

### Feature selection results

A set of features were selected from a total of 89 features through IBCGA. Then, the ensemble classifier with 31 stable models with different feature combinations was established, and the final model predicted the answer of the LN types by voting on 31 models. In the 31 models, Gray-Level features were the most frequently selected subset features, followed by Edge features, Sum of variance, and 3D morphology features. Among them, 3D morphology features were mainly suitable for distinguishing ENE LNs.

Each of the 31 models had a satisfactory prediction ability. From the analysis of the subset features of the 31 models, the features that were selected more than 16 times represent that they had a significant influence on the voting process and were the most influential. The top-rank features in the best combination feature set were shown in Table 3. The GLSZM subset feature contained Low intensity small area emphasis, Zone%, High intensity large area emphasis, and small area emphasis. Among them, small area emphasis had the smallest p-value, 3.454e-09.

**Table 3:** The selection times of features in the ensemble classifier consisting of 31 SVMs

Subset feature	Feature name	Times of selection
GLSZM	Low intensity small area emphasis	31
3D morphology	Orientation2	30
GLSZM	Zone%	29
GLSZM	High intensity large area emphasis	29
Grey level	Median	28
Grey level	Max Pixel Value	28
3D morphology	Orientation3	27
Grey level	Variance	26
GLSZM	small area emphasis	24
3D morphology	Solidity	21
3D morphology	Max radius	19
3D morphology	Area	18
Grey level	Energy	17
Sum of variance	D1A45	16
3D morphology	Compactness	16

Four Grey-level subset features were selected, including Median, Max Pixel Value, Variance, and Energy, where Variance had the smallest p-value, 1.821e-18. The normal LNs had the most significant value of Variance, and ENE LNs had the smallest value of Variance. In addition, the same results were found in the analysis of D1A45 (distance 1, direction 45 degrees) in Sum of variance of GLCM.

The difference between the small area emphasis, Variance, and Sum of variance D1A45 was that the small area emphasis focused on the grey level change related to the size of the area changed, the Variance focused on the grey level change of the entire image, and the Sum of variance D1A45 focused on the grey level change in specific distances and angles.

**Fig. 2:**
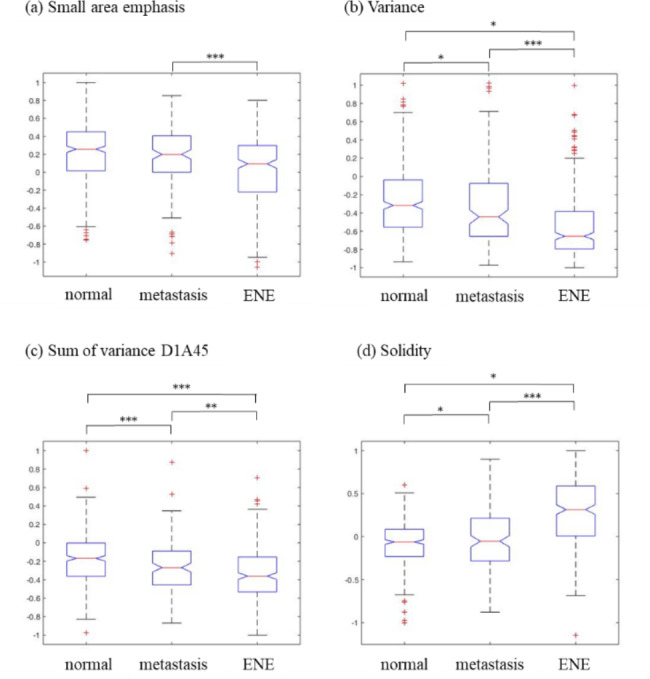
The boxplots of (**a**) Small area emphasis, (**b**) Variance, (**c**) Sum of variance D1A45, and (**d**) Solidity in the normal, metastatic, and extranodal extension lymph nodes

Six 3D morphology subset features were selected, including Orientation2, Orientation3, Solidity, Max radius, Area, and compactness. Solidity had the smallest p-value, 7.182e-42. Solidity represented the irregularity of the surface. The boxplot of the small area emphasis, Variance, Sum of variance D1A45, and Solidity in three types of LNs were shown in Fig. 2.

**Fig. 3:**
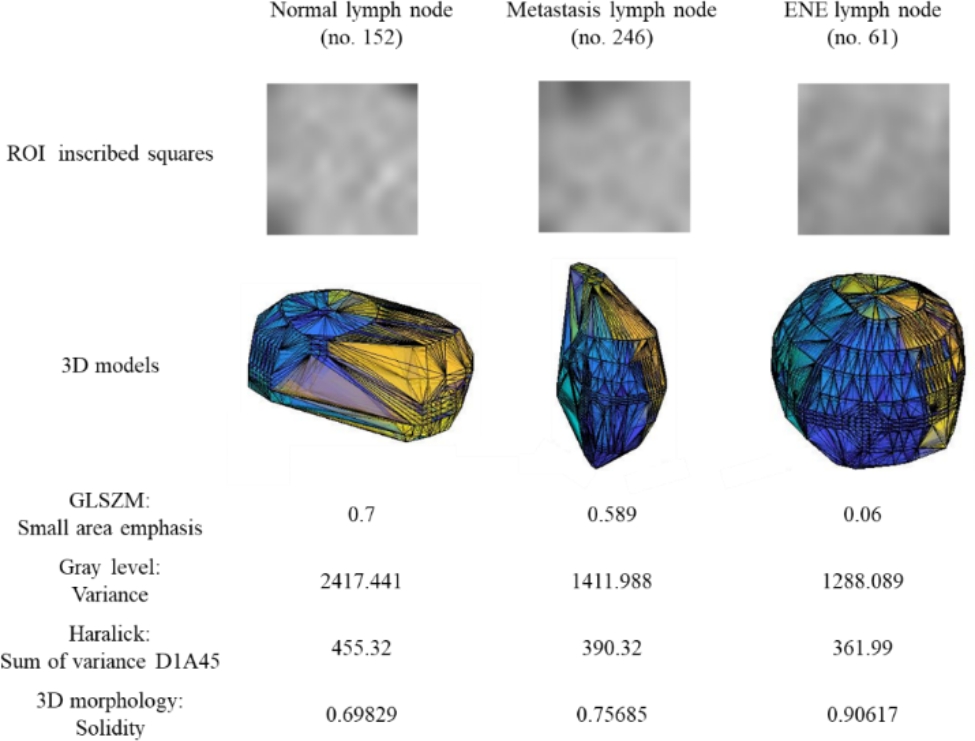
The regions of interest inscribed squares and 3D models of normal lymph nodes (no. 152), metastatic lymph nodes (no. 246), and ENE lymph nodes (no. 61)

Figure 3 showed the inscribed squares in ROI and 3D models of the three types of LNs, including normal LNs (no. 152), metastatic LNs (no. 246), and ENE LNs (no. 61). Although it was difficult to distinguish the difference in texture with the human eye [30], the analysis revealed valuable information that normal LNs have the largest values of the small area emphasis, Variance and Sum of variance D1A45. For the 3D features, normal LNs had the smallest value of Solidity, and ENE LNs had the most significant value of Solidity.

### Prediction performance of EL-ENE model and radiologists

The EL-ENE method established 31 independent prediction models. All the results were counted, the voting method was adopted, and the final answer was decided by a majority. The EL-ENE ensemble model was trained by 618 LNs and independently tested by 160 LNs.

**Fig. 4:**
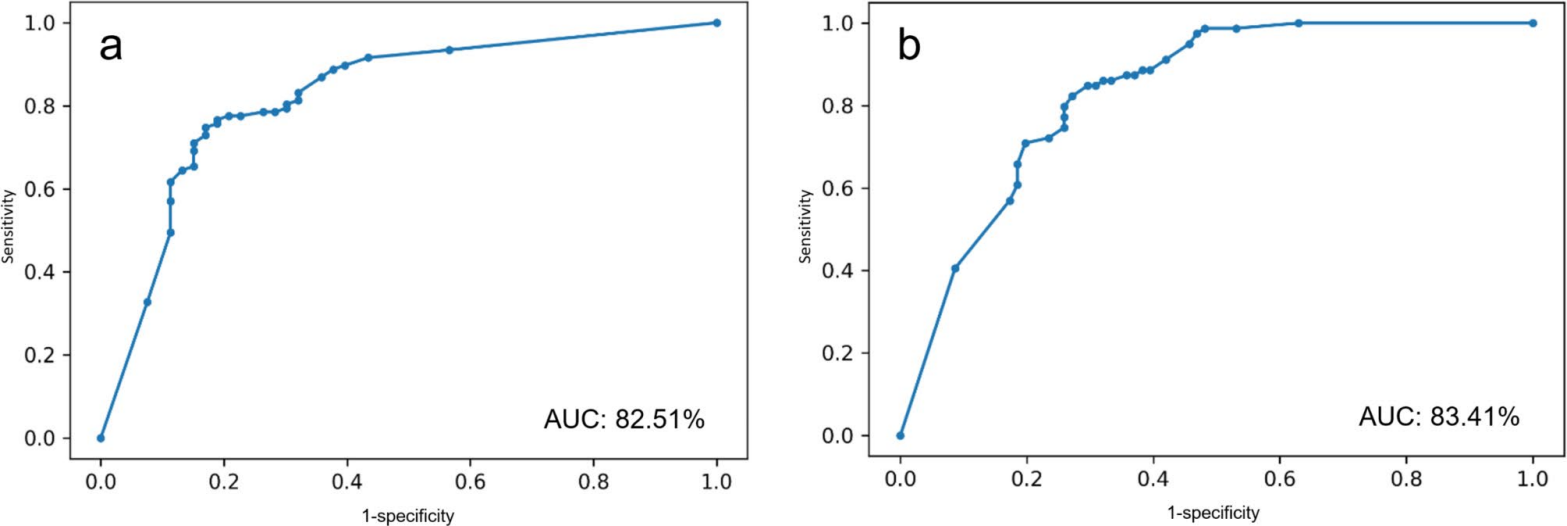
The receiver operating characteristic curve of extranodal extension prediction and nodal metastasis prediction models on an independent test set

The EL-ENE ensemble model achieved test accuracy of ENE prediction 80.00%, sensitivity 81.13%, specificity 79.44%, PPV 66.15%, NPV 90.32%, and AUC: 82.51%. For metastasis prediction, the prediction model achieved accuracy 77.50%, sensitivity 70.37%, specificity 84.81%, PPV 82.61%, NPV 73.63%, and AUC 83.41%. (Table 4; Fig. 4)

The radiologists’ prediction performance for ENE prediction achieved test accuracy 70.44%, sensitivity 75.64%, specificity 67.91%, PPV 50.04%, NPV 85.43%, and AUC 71.78%. For metastasis prediction, the prediction performance was as follows: accuracy 73.79%, sensitivity 76.54%, specificity 70.94%, PPV 74.53%, NPV 74.67%, and AUC 71.87%. The EL-ENE model performs significantly better prediction performance than two of the radiologists (p-value = 0.0006 and 0.002), and no statistically significant difference with the third radiologist (p-value = 0.654). (Table 4)

**Table 4:** The prediction performance of the EL-ENE model and radiologists

	EL-ENE model				Radiologists	
	ENE Prediction		Metastatic Prediction		ENE Prediction	Metastatic Prediction
	Training Set	Test Set	Training Set	Test Set	Test Set	Test Set
Accuracy	82.52%	80.00%	78.96%	77.50%	70.44%	73.79%
Sensitivity	79.15%	81.13%	66.77%	70.37%	75.64%	76.54%
Specificity	84.27%	79.44%	92.23%	84.81%	67.91%	70.94%
PPV	72.81%	66.15%	89.92%	82.61%	50.04%	74.53%
NPV	88.46%	90.32%	71.58%	73.63%	85.43%	74.67%

## Discussion

We have proposed an evolutionary learning method for establishing a transparent and interpretable ensemble classifier to predict metastatic and ENE LNs in HNSCC patients. This model shows superior classification ability to the radiologists while providing exquisite interpretable information to physicians. Many selected radiomics features can find reasonable clinical or pathological relevance. For example, small area emphasis, the most popular feature selected in the classification model, may represent the invasion of tumor cells and necrotic changes in a metastatic or ENE LN [31]. A larger small area emphasis value means a finer texture in the small area. In our data, normal LNs possess the largest value of small area emphasis. As the pathological changes of cancer cell invasion and necrosis development progress, this value decreased in the metastatic LNs and became the smallest value in the ENE LNs. Our study also finds that the 3D morphology features, which are rarely mentioned in published literature, are powerful for detecting ENE LNs. These implicit and subtle features may provide further clinical insights for ENE image evaluation in the future. The results revealed that an interpretable model can not only provide excellent prediction ability but also correlate the association between the radiomics features and novel clinical knowledge.

Interpretability is critical for clinical prediction models. Understanding the correlation between the input data, the prediction results, and the principles of decision-making behind the EL algorithms may gain the trust and confidence of the prediction models to the clinical practitioners [32] because clinical decision-making is based on logical reasoning, rigorous inference, and solid evidence [33–35]. Due to the lack of interpretability, clinicians may be more conservative in applying black-box algorithms to support clinical decision-making, especially in the high-stake clinical scenarios [36]. It is also challenging to detect or even be aware of potential model errors or biases in an opaque prediction model [37]. Furthermore, an interpretable prediction model might discover comprehensible novel information for future clinical practice [38]. That is to say, clinicians may learn new knowledge from the interpretable models by analyzing their “thinking process”. Consequently, interpretable EL-based medical applications are more trustworthy, robust, creative, and more feasible for clinical practice.

Deep learning has been heavily applied to medical image research for constructing appealing high-accuracy diagnostic and prediction models in individual studies in recent years [39–45]. In ENE detection, Kann and his colleagues developed the first deep learning 3D convolutional neural network model with impressive diagnostic performance and comprehensive external validation [15, 46, 47]. Both this deep learning model and our EL-ENE model outperformed most radiologists in ENE detection. Their AUCs are numerically higher than our model, although we cannot directly compare results from different data sets. Deep learning algorithms such as convolutional neural networks can automatically and adaptively learn complex imaging features and establish sophisticated models [48]. Therefore, with sufficient data, these models might catch essential features beyond the pre-defined radiomic features and potentially achieve better prediction outcomes.

However, the widespread adoption of deep learning models into daily clinical practice is yet to be established [49]. One major reason for this disproportionate phenomenon is that the data-driven nature of deep learning models is often referred to as black-box algorithms [40]. Therefore, most early deep learning applications have the inherent shortcomings of intransparency and uninterpretability. These model defects may erode the physicians’ confidence in deep learning models and further restrict the wide acceptance of these models into clinical practice. Recently, there has been increasing research on interpretable deep learning models to mitigate the opacity and uninterpretability of deep learning models [50, 51]. Various methods have been developed for building more interpretable deep learning models with promising results [51]. With the rapid progress of interpretable ML, a more comprehensive deep learning algorithm might create more trustworthy prediction models and increase the adoption of its applications into clinical practice in the future.

The diagnostic and prediction power of ML models is not always unlimited. In our case, the physical limitations of the diagnostic CT images may restrict its accuracy for recognizing metastatic or ENE LNs. For example, the z-axis resolution in standard diagnostic helical CT images with 2–3 mm slice thickness may not be sufficient to identify subtle micro ENE [52]. Moreover, uncertainties from CT homogeneity, Hounsfield number accuracy, image linearity, noise interference, and artifact may further hamper the diagnostic ability of CT images [53]. Therefore, if a ML model provides exaggerated results beyond our expectations, we should carefully examine that model for potential errors or biases. Undoubtedly, an interpretable ML model is also more applicable for this purpose.

This study has several limitations. First, all images were collected in a single institution. The generalizability of this model should be further validated. Second, some LN data were discarded during data collection because a definite correlation between CT images and pathology reports could not be established. Finally, the CT slice thickness is 2–3 mm which may limit the special resolution. Some subtle image features might be blurred due to this relatively thick CT slice thickness.

Future research is warranted to overcome the above limitations. First, external validation is essential for evaluating model generalizability and the robustness and consistency of selected radiomics features. This process could further strengthen the reliability of this explainable EL-ENE model and increase confidence in applying this model in clinical practice. Second, modern medical imaging, such as high-resolution CT or magnetic resonance imaging, might further improve the model’s performance. These advanced medical images possess more clinical information and better resolution for discriminating subtle image features such as micro ENE. With a similar model build-up process, we can build an enhanced EL-ENE model with these modern medical images with potentially better performance.

## Conclusions

In addition to the pursuit of accurate ENE prediction models, a transparent ML algorithm may provide more comprehensible and robust models for medical applications. Furthermore, these models may explore novel features to expand our clinical knowledge. We believe that more clinicians will be pleased to adopt these trustworthy applications into their daily practice in the future.

### Electronic supplementary material

Below is the link to the electronic supplementary material.

Supplementary Material 1

## Data Availability

The datasets generated during and/or analyzed in the current study are available from the corresponding authors upon reasonable request.

## List of abbreviations

10-CV: 10-fold cross-validation
AUC: Area under the receiver operator characteristic curve
CT: Computed tomography
DICOM: Digital imaging and communications in medicine
EL: Evolutionary learning
ENE: Extranodal extension
HNSCC: Head and neck squamous cell carcinoma
IBCGA: Inheritable bi-objective combinatorial genetic algorithm
IEA: Intelligent evolutionary algorithm
LN: Lymph node
ML: Machine Learning
ROI: Regions of interest
RTSS: RT structure set
SVM: Support vector machine
VOI: Volume of interest
