# Supplementary material for: Prediction of extranodal extension in head and neck squamous cell carcinoma by CT images using an evolutionary learning model
Source: Cancer Imaging. 2023 Sep 12;23:84. doi: 10.1186/s40644-023-00601-7 (PMC10496246; doi:10.1186/s40644-023-00601-7)
Supplement: Supplementary file 1 — Supplementary Material 1 [file 40644_2023_601_MOESM1_ESM.docx]

**Appendix**

Table A1: The top 5 10-fold validation accuracy result of the subset features in metastatic prediction. Normal LNs were classified as class 1 and metastatic LNs and ENE LNs were classified as class 2.

| Subset feature name | Training set accuracy | Training set MCC | 10-fold cross validation accuracy | 10-fold cross validation MCC |
| --- | --- | --- | --- | --- |
| 20. Gray level | 77.994% | 0.610 | 70.712% | 0.501 |
| 25. Edge 10 | 71.521% | 0.515 | 68.123% | 0.466 |
| 17. Sum Variance | 71.359% | 0.507 | 67.638% | 0.455 |
| 21. 3D Morphology | 68.932% | 0.484 | 67.476% | 0.461 |
| 24. Edge 5 | 71.359% | 0.511 | 67.314% | 0.454 |

Table A2: The top 5 10-fold validation accuracy result of the subset features in ENE prediction. Normal LNs and metastatic LNs were classified as class 1 and ENE LNs were classified as class 2.

| Subset feature name | Training set accuracy | Training set MCC | 10-fold cross validation accuracy | 10-fold cross validation MCC |
| --- | --- | --- | --- | --- |
| 21. 3D Morphology | 78.803% | 0.537 | 77.508% | 0.508 |
| 20. Gray level | 79.450% | 0.563 | 76.375% | 0.505 |
| 24. Edge 5 | 76.052% | 0.497 | 75.243% | 0.482 |
| 19. GLSZM | 82.524% | 0.623 | 72.816% | 0.438 |
| 25. Edge 10 | 74.272% | 0.462 | 72.006% | 0.419 |

Table A3: The top 5 10-fold validation accuracy result of the subset features in three classification prediction. Normal LNs were classified as class 1, metastatic LNs were classified as class 2, and ENE LNs were classified as class 3.

| Subset feature name | Training set accuracy | 10-fold cross validation accuracy |
| --- | --- | --- |
| 21. 3D Morphology | 70.874% | 61.812% |
| 20. Gray level | 77.67% | 61.327% |
| 23. Edge 3 | 61.812% | 61.003% |
| 17. Sum Variance | 62.46% | 60.518% |
| 25. Edge 10 | 61.812% | 60.194% |
